# Myocarditis and Myocardial Disease—IV

**Published:** 1908-08-29

**Authors:** 


### Medicine.

### MYOCARDITIS AND MYOCARDIAL DISEASE—IV.

### VIII. Fatty Degeneration.

Fatty degeneration of the heart differs entirely from fatty infiltration, in that the lesion lies in the muscle fibres themselves and not between them. The sarcous elements gradually become replaced by fatty granules and droplets, the change beginning about the nuclei and extending linearly towards the ends of the muscle cells. The affected tissue is thus rendered paler and more opaque than normal, in streaks of a tawny yellow colour. In chronic cases the change often appears in patches, and gives rise to a yellowish mottling of the tissue, generally most marked on the inner surface of the heart-wall, the trabeculæ, and the papillary muscles, especially in the left ventricle. The mottling often recalls the appearance of the grain of some fine cabinet wood, or the pattern of a delicately striated feather; the terms usually applied to it are "tabby cat" or "throstle's breast" striation. It should be noted, however, that the microscope may reveal extensive fatty degeneration in cases in which the heart muscle exhibits no definite abnormality to the naked eye.

The change may take place almost acutely in conditions of prolonged bacterial toxæmia, and as the result of poisoning by arsenic, lead, phosphorus, carbon monoxide, or antimony. The most decided examples are seen in chronic profound anæmias, especially pernicious anæmia.

### IX. General Interstitial Fibrosis—Fibroid Heart.

The essential lesion in a fibroid heart is an increase in the interstitial connective tissue throughout the organ, seldom uniformly distributed, but nearly always exhibiting pale bands and streaks in the cross-section corresponding to the places where the fibrosis has been most extensive. The change is not itself an inflammation, though it is often spoken of as "chronic myocarditis." It is in most instances the result of an effort at repair; there may be an inflammatory element preceding the fibrosis, as will be described below, but the inflammation and the fibrosis are not to be regarded as one and the same thing. The change is one of gradual progress and slow accumulation, with the result that in nearly every instance the heart is big; it is sometimes very big. The symptoms are those common to all forms of chronic heart disease, and the diagnosis is often guess-work. If one point more than another attracts one's attention to the nature of the lesion it is that angina pectoris is sometimes a prominent symptom. If there is no evidence of aortic or mitral valvular disease, if the heart is very big without obvious cause, such as granular kidney, if the patient is middle aged or older, and if angina pectoris is a prominent symptom, a diagnosis of cardiac fibrosis will probably be right if the patient is a thin man, whilst if he is very stout the diagnosis will lie between fibrosis and fatty infiltration of the heart.

There have been many different classifications of the causes of general cardiac fibrosis, but it seems most useful to keep the headings down to as small a number as possible, in which case one may include the origins of almost all fibroid hearts under two main headings—namely, (1) Syphilis and (2) obstruction to the coronary circulation. Syphilis comes in under both these headings, for it may cause atheroma of the coronary arteries under heading (2); and it may also lead to a very diffuse small round-celled infiltration of the whole of the interstitial tissue of the heart, probably of the nature of multiple small gummata.

Apart from diffuse syphilitic fibrosis of this kind, however, almost all cases of fibroid heart may be traced in their origin either to obstruction to the blood reaching the muscle by the coronary arteries, or to obstruction to the blood leaving the muscle by the coronary veins. In the first category come all the causes of atheroma and arterio-sclerosis, so that fibroid heart may result from simple old age, premature or otherwise, syphilis, chronic alcoholism, arterio-sclerosis, severe and hard work under unhealthy conditions, high living, gout, and plumbism; in the second category come all the causes that, acting over long periods, may delay the return of the blood into the right auricle, especially chronic pulmonary fibrosis with or without bronchiectasis, emphysema with persistent bronchitis, asthma, chronic valvular heart disease, adherent pericardium, and so forth.

If we are to understand the process by which similar cardiac fibrosis arises from so many causes which at first sight seem to have little in common, one must return to the subject of myomalacia cordis, described in Article II., and inquire what happens to the ischæmic patches in patients who do not die of them in the acute stage. The tissue changes underlying the varying appearances of the softened patches are partly retrogressive and partly constructive in their character. The original ischæmia brings about the destruction of numbers of muscle cells whose fragments ultimately break down into granular detritus. In the case of quite small lesions, after the disintegration of the muscle-cells, the process may come to an end; in other cases further changes take place in the connective-tissue elements. Inflammatory exudations of small round cells very soon supervene around the areas affected by ischæmia, and regenerative processes start from the connective tissue of the neighbouring parts. The proliferation leads to the formation of granulation tissue, with the production of new vessels and white fibres, while the products of disintegration are partly dissolved and partly taken up by the cells themselves. Presently the proliferation of the small round cells ceases, the granulation tissue becomes more and more mature, until its place is occupied by a band, or streak, or cicatrix of fibroid induration or sclerosis.

Small areas of ischæmia naturally leave behind them small patches of sclerosis which lie hidden in the muscle, giving rise to no perceptible thinning of the wall, and causing no important disturbance of the function of the heart. They become of importance when, later, there is an accumulation of similar small areas of fibrous tissue throughout the heart.

